# Institutional barriers to advancement in academic hospital medicine: A perspective from the margins

**DOI:** 10.1002/jhm.70159

**Published:** 2025-08-16

**Authors:** Hafiz Qurashi

**Affiliations:** ^1^ Division of General Internal Medicine, Section of Hospital Medicine UPMC Presbyterian and Montefiore Hospital Pittsburgh Pennsylvania USA

It was 1:45 p.m., and I had just returned from my rounds and was completing notes after a demanding day managing complex transplant patients. A senior colleague, well‐respected at our institution, entered the workroom and asked if I would consider applying for an open administrative position on our team. He explained the scope, agreed it suited my goals and skills, encouraged me to apply, and mentioned that an announcement would be sent out shortly.

When the email arrived, I replied immediately, expressing genuine excitement and interest. I returned to clinical care feeling hopeful that this could mark a new chapter in my academic journey. Weeks later, I received a brief reply: “Thank you for your interest. After discussions among the leadership, we have decided to proceed with another candidate.” No feedback. No stated selection criteria. No context.

I can't claim certainty about why I wasn't selected. In the absence of feedback or transparent criteria, however, I'm left to wonder whether my status as an international medical graduate (IMG) played a role. That doubt isn't rooted in a single experience; it reflects a broader pattern. Colleagues with similar training backgrounds have described near‐identical scenarios: roles that quietly disappear after interest is expressed, feedback that never arrives, or opportunities extended only to those with more conventional pedigrees. These moments accumulate not as isolated disappointments, but as signs of a system that remains opaque. What's often interpreted as a lack of readiness or fit may instead reflect a lack of access, sponsorship, or familiarity with institutional norms—barriers that are invisible, but no less real.

As a hospitalist who trained in the United States after completing medical school abroad, I have spent nearly a decade building a career in academic hospital medicine. Like many IMGs, I arrived without an established network or institutional pedigree. I volunteered for educational projects, collaborated on research, and embraced every role offered. Professional advancement has often felt like navigating a landscape without markers—where the path forward is not only unclear but shaped by invisible boundaries and shifting expectations. Colleagues at my institution and across the country with similar training backgrounds have echoed these experiences. Many are high‐performing clinicians and educators who find themselves bypassed for advancement, unsure whether the gap lies in credentials, perception, or simply access to the right rooms.

These anecdotes reflect the well‐documented, complex, and multifactorial nature of bias in academic medicine. Even groups that are not underrepresented in medicine overall, such as Asian American physicians, have been shown to face barriers to advancement and leadership roles despite strong pipeline representation.^1^ Similarly, while gender equity has been the focus of sustained efforts and data collection, women now comprise approximately 50% of US medical school graduates but hold only 27% of dean positions.^2^ These examples illustrate how inequities can persist despite recognition and intervention, underscoring the need for the same level of scrutiny, data, and transparency in advancement pathways for IMGs. In one national survey, over 70% of residency program directors acknowledged that IMGs face discrimination during the selection process.^3^ That perception likely extends into the faculty pipeline. Even as IMGs contribute substantially to the care of the underserved and health workforce diversity, they remain underrepresented in leadership roles at academic medical centers.^4^, ^5^

This is not a plea for special treatment. IMGs are held to the same licensure and credentialing standards, and in many cases, exceed them. The issue is not ability but visibility and recognition. Advancement in academic hospital medicine should depend on capability and contributions—clinical excellence, educational impact, and scholarly work—not birthplace or pedigree. Yet current systems often lack transparency, which allows implicit bias or differences in cultural familiarity with institutional expectations to influence advancement opportunities. These include unwritten norms around speaking up in meetings, advocating for one's contributions, or navigating informal networks—practices that may differ across training cultures, particularly where deference, restraint, or indirect communication are the default. Such differences, while rarely acknowledged, can meaningfully influence how potential is perceived within the academic environment.

The structural dynamics of academic hospital medicine make this even more salient. Hospitalists frequently serve on the front lines of care delivery and education; yet, the pathway from clinical roles to leadership often hinges on informal sponsorship, insider knowledge, or existing networks—elements that disproportionately disadvantage those from nontraditional backgrounds. The result is not just individual disappointment, but a systemic loss, as diverse perspectives and talent remain underleveraged.

Institutions must critically examine how leadership roles and promotions are assigned. This includes establishing clear and transparent criteria for advancement, offering direct and actionable feedback to applicants regarding qualifications or expectations, and reviewing advancement processes to ensure equity. Such feedback is especially important because, without transparency, applicants may find it difficult to understand whether decisions are based on their skills, experience, and cultural fit, or whether they are driven by systemic bias. Inclusion efforts are ineffective if the decision‐making remains opaque or selection criteria are inconsistently applied. Addressing procedural ambiguity is essential, and just as important is recognizing how individual bias can shape those decisions. Fostering bias‐awareness among decision‐makers is a key complement to structural reforms. Recommended strategies—such as structured reflection, implicit bias workshops, and institutional introspection can help individuals recognize and begin to address how unconscious preferences influence advancement decisions.^6^ Institutions need to ask themselves: Do our promotion practices truly reflect our core values of diversity, equity, and inclusion?

After receiving that rejection email, I returned to what I do best, caring for patients, mentoring residents, and contributing to the broader academic mission. However, the discomfort lingered, not as a personal grievance but as a prompt to reflect on broader patterns. The lack of clear criteria, the silence after decisions, and subtle patterns of exclusion are not anomalies; they are indicators of a system that still needs reform. What we need is not an exception but a sustained commitment to equity by creating systems where merit is recognized, feedback is provided, and advancement is truly accessible to all hospitalists, regardless of background.

## CONFLICT OF INTEREST STATEMENT

The author declares no conflicts of interest.

## References

[1] Choi AMK , Rustgi AK . Diversity in leadership at academic medical centers: addressing underrepresentation among Asian American faculty. JAMA. 2021;326(7):605‐606. 10.1001/jama.2021.12028 34402820

[2] Lautenberger DM , Dandar VM . Association of American Medical Colleges. State of Women in Academic Medicine: 2023–2024 Progressing Toward Equity. Association of American Medical Colleges; 2024. Accessed July 8, 2025. https://www.aamc.org/data-reports/data/state-women-academic-medicine-2023-2024-progressing-toward-equity

[3] Desbiens NA , Vidaillet HJ . Discrimination against international medical graduates in the United States residency program selection process. BMC Med Educ. 2010;10(1):5. 10.1186/1472-6920-10-5 20100347 PMC2822781

[4] Zaidi Z , Dewan M , Norcini J . International medical graduates: promoting equity and belonging. Acad Med. 2020;95(12S):S82‐S87. 10.1097/ACM.0000000000003694 32889932

[5] Smith SM , Parkash V . Normalized “medical inferiority bias” and cultural racism against international medical graduate physicians in academic medicine. Acad Pathol. 2023;10(4):100095. 10.1016/j.acpath.2023.100095 37767366 PMC10520300

[6] Marcelin JR , Siraj DS , Victor R , Kotadia S , Maldonado YA . The impact of unconscious bias in healthcare: how to recognize and mitigate it. J Infect Dis. 2019;220(suppl 2):S62‐S73. 10.1093/infdis/jiz214 31430386

